# Diagnosis and treatment of malignant retroperitoneal mesothelioma: A case report

**DOI:** 10.1097/MD.0000000000037985

**Published:** 2024-04-26

**Authors:** Feihu Tang, Yong Cui, Yuan Gao

**Affiliations:** a Department of Clinical Medicine, Shandong Second Medical University, Weifang City, Shandong, China; b Department of Urinary Surgery, Weifang People’s Hospital, No.151 Guangwen Street, Weifang City, Shandong, China.

**Keywords:** immunotherapy, retroperitoneal mesothelioma, tumor cell reduction

## Abstract

**Rationale:**

Malignant peritoneal mesothelioma (MPM) is a rare clinical disease. Although there are several reports describing intraperitoneal mesothelioma of the lung, liver, and intestine, retroperitoneal mesothelioma is, to our knowledge, very rare and rarely reported. In recent years, our best clinical protocols for the treatment and diagnosis of retroperitoneal mesothelioma have not been proven and the diagnosis and treatment are challenging.

**Patient concerns:**

A 37-year-old Chinese woman complained of bilateral low back pain for a month, with obvious symptoms of low back pain on the left side. To treat low back pain, retroperitoneal masses were found during physical examination. The patient consulted a urological specialist for further treatment.

**Diagnosis:**

After the operation, pathological biopsy confirmed retroperitoneal epithelioid diffuse mesothelioma.

**Interventions:**

After exclusion of surgical contraindications, the patient underwent laparoscopic retroperitoneal lesion resection under tracheal intubation and general anesthesia, and the operation was successful.

**Outcomes:**

On the tenth day after surgery, the patient vital signs were stable, and he was discharged.

**Lessons:**

Patients with malignant peritoneal mesothelioma may have no typical clinical symptoms, and the diagnosis is based on pathological and immunohistochemical examination. In selected patients, surgical cell reduction and intraoperative intraperitoneal heat chemotherapy have become the first choice of treatment, which can achieve ideal therapeutic effects and prolong survival.

## 1. Introduction

Malignant peritoneal mesothelioma (MPM) is a very rare, progressive, and ultimately fatal serous malignancy.^[1]^ It is reported that the annual incidence of MPM in Europe is about 1 to 2 million, the number of new cases in the United States is about 250 per year,^[2]^ and the case of retroperitoneal mesothelioma is even less. The most common type of malignant mesothelioma is pleural malignant mesothelioma, MPM accounts for almost 12.5% to 25% of all mesothelioma diagnoses,^[3]^ and the proportion of MPM in retroperitoneal malignant mesothelioma is unclear because it is very rarely reported. There are 3 histological subtypes of MPM: epithelioid MPM accounts for about 70% to 80%, and sarcomatoid MPM and biphasic MPM account for a lesser proportion.^[4]^ Asbestos exposure is a major contributor to MPM, however, there are cases in which there is no history of asbestos exposure.^[5]^ Diagnosis and treatment of peritoneal mesothelioma are often delayed due to nonspecific and heterogeneous clinical symptoms. In addition, the prognosis for peritoneal mesothelioma is poor, with a median survival of 12 months for patients with malignant mesothelioma, so early diagnosis and treatment initiation are critical.^[6]^ Here, we present a case report on a patient with malignant retroperitoneal mesothelioma.

## 2. Case presentation

**Figure 1.**
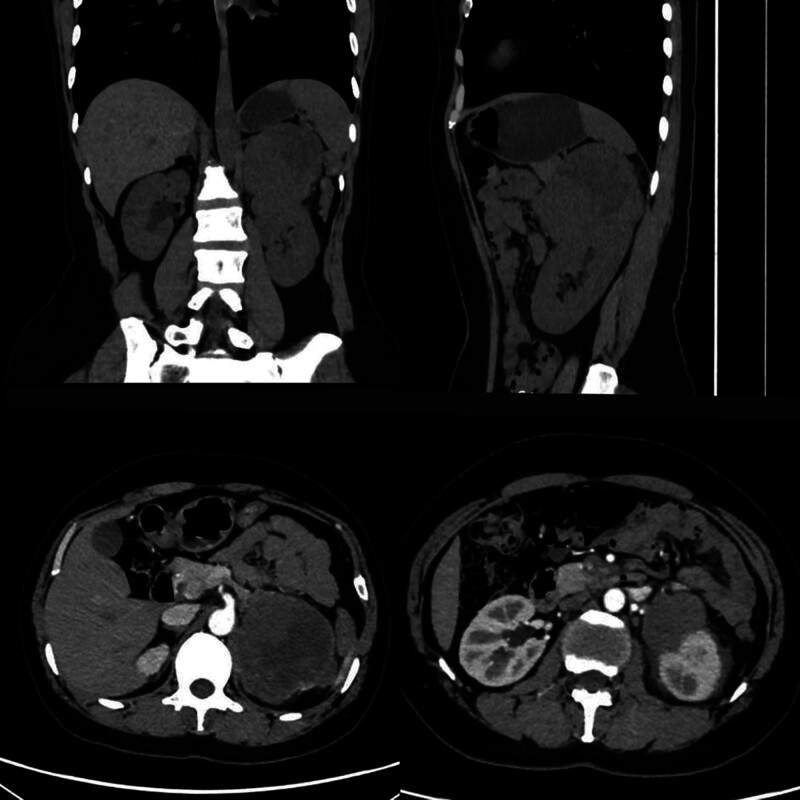
An irregular soft tissue density shadow was found in the left adrenal area, with clear boundaries and uneven density, about 10.4 × 7.4 cm in size. The enhanced scan showed mild uneven enhancement, and small blood vessels were seen inside. The left adrenal gland is unclear, and the local boundary between the lesion and the left kidney is unclear. Small lymph node shadow can be seen retroperitoneally.

The patient was a 37-year-old middle-aged female who complained of bilateral low back pain for a month, with obvious symptoms of low back pain on the left side. Retroperitoneal masses were found during physical examination for the treatment of low back pain, without abdominal pain, distension, frequent urination, and other discomfort. The patient came to the hospital for further treatment. After admission, relevant examinations were completed. Abdominal CT plain scan plus enhanced scan (Fig. 1) indicated that the left adrenal area was occupied, and retroperitoneal lymph nodes were occupied. The 6 values of ACTH, CA125, and sex hormones were all normal (The results of ACTH and CA125 were 15.050 ng/L and 21.71 U/mL, respectively. Among the sex hormones, the results of progesterone were 13.09 nmol/L, estradiol 361.30 pmol/L, prolactin 463.30 μIU/Ml, luteinizing hormone 7.86 IU/L, follicle-stimulating hormone 5.84 IU/L and testosterone 0.87 nmol/L).

**Figure 2.**
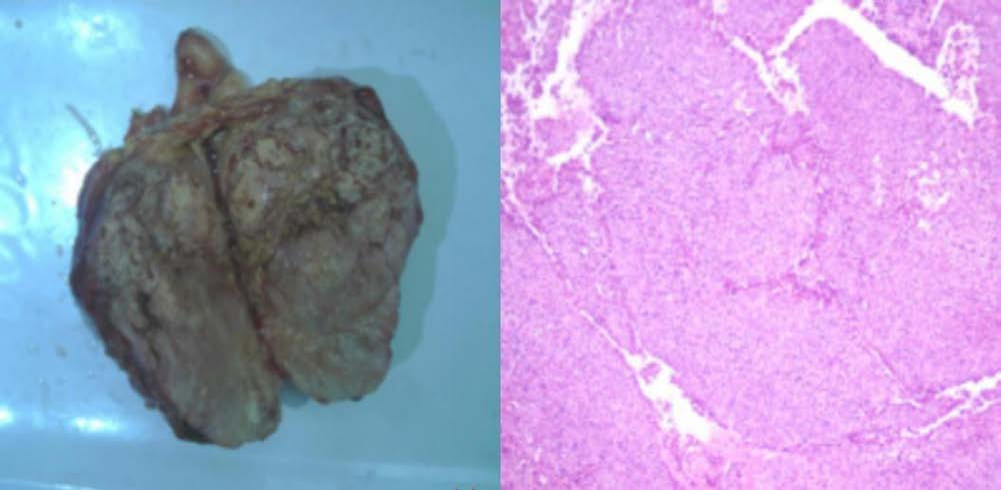
General observation:(left retroperitoneal tumor) a nodular object with a volume of 10.5 cm × 8 cm × 6.5 cm was attached to the surface of the capsule, which was sallow and medium in thickness, and locally necrotic. Adjacent to the capsule, part of the adrenal gland was attached, 8 cm × 6 cm in area, 0.2 cm to 0.5 cm thick, and the section was sallow and medium in quality. There were several palpable lymph nodes (left retroperitoneal lymph nodes) with a length of 0.5 cm to 1.2 cm.

After the patient contraindications were excluded, laparoscopic retroperitoneal lesion resection (left) was performed under tracheal intubation and general anesthesia. The surgical procedure was as follows: After successful anesthesia, with the assistance of laparoscopy, the perirenal fascia was opened, and when the left kidney was freed to the upper extremity of the left kidney, a hard mass with a diameter of about 11 cm and some surface cystic was found. The blunt peel along the surface of the tumor was continued upward to free the renal artery, and multiple enlarged lymph nodes were seen on the surface of the abdominal aorta. The lymph nodes were carefully swept along the abdominal aorta. Continuing to isolate the tumor, the tumor involved the renal capsule and adrenal gland. The involved renal capsule and adrenal gland were removed together, and the operation was successful. Postoperative pathology (Fig. 2) Results: (left retroperitoneal fat tumor) epithelioid malignant tumor, was epithelioid diffuse mesothelioma with rhabdomyoid structure, local invasion of the adrenal gland with adrenal compression atrophy: tumor metastasis was found in the lymph node (left retroperitoneal 2/20), and multiple ganglion cell neuromas were also observed. Immunohistochemical results: Wax block Number: S2318828-A4: + Desmin (part), C10 (+), D2-40 + (part), CD31 (vascular +) and CD34 + (blood vessels), ERG (−), PR (−), RCC (−), CAIX part (+), SMARCA4 (+). INI1 part (+), My0D1 (−), Actin (−), T TF-1 (−), CD138 part (+), S MA (−), Calponin (−), S0X10 (−), the BCL-2 (−), CD21 (−). CD23 (−). CD68 (−), CD38 (−), LCA (−). Special staining results, wax block No.: S2318828-A4: reticular fiber (−), AB/PAS (−).

## 3. Discussion

MPM symptoms are related to the extent to which the tumor has spread within the abdominal cavity; the most common symptom reported in the literature is abdominal pain, followed by weakness, weight loss, anorexia, abdominal mass, fever, diarrhea, and vomiting.^[7]^ This patient is retroperitoneal malignant mesothelioma, and there are no intraperitoneal symptoms as in MPM patients. The main manifestation of this patient is lumbar pain caused by retroperitoneal space occupation.

In terms of imaging, typical CT findings of peritoneal mesothelioma include heterogeneous solid mass with irregular edges, ascites, greater omental involvement, and peritoneal thickening, etc, but there are no typical imaging features, and MRPM is rare in clinic, so it is impossible to make a diagnosis based on imaging findings alone. Early diagnosis is often very difficult, so patients may delay treatment and affect prognosis. In this case, malignant retroperitoneal mesothelioma only showed irregular soft tissue density shadows in the adrenal area and small retroperitoneal lymph node shadows on CT, with no characteristic imaging findings. MRPM can be biopsied by pathology and immunohistochemistry to provide a definitive diagnosis. In particular, the recently introduced immunohistochemical marker BRCA1-associated protein 1 is also particularly useful in determining whether mesothelial cells in cytological specimens are malignant or benign, and germline mutations in BRCA1-associated protein 1 increase susceptibility to mesothelioma.^[8]^

The gold standard of treatment remains cell reduction (CRS) combined with intraperitoneal hyperthermic chemotherapy (HIPEC),^[9]^ which extends overall survival from a median of 6 months in primary patients to 34 to 92 months in patients with CRS and HIPEC.^[10]^ Systemic chemotherapy is usually used in patients who are not candidates for surgical treatment or who are at high risk of early recurrence after tumor cell reduction. Pemetrexed combined with platinum-based drugs (cisplatin or carboplatin) is currently the recommended systemic chemotherapy regimen worldwide.^[11]^ At present, the targeting pathway of MPM is being identified. ALK rearrangement in a small number of patients recently reported can often benefit from ALK inhibitor therapy.^[12]^

## 4. Conclusion

Retroperitoneal mesothelioma is a very rare malignant tumor with few cases reported in the previous literature, and the diagnosis and treatment of this malignant tumor are challenging. Although current treatment therapies have improved survival, the prognosis remains poor. More clinical studies are needed to standardize the treatment of MPM, and the combined use of checkpoint blockade with surgical resection or local intracavitary immunotherapy deserves further investigation. The purpose of this case report is to improve the understanding of malignant retroperitoneal mesothelioma and to find a more effective treatment. In this way, the correct treatment method can be selected before surgery to achieve the ideal therapeutic effect.

## Author contributions

**Conceptualization:** Yuan Gao.

**Data curation:** Yong Cui.

**Methodology:** Feihu Tang.

**Supervision:** Yuan Gao.

**Validation:** Yong Cui.

**Writing – original draft:** Feihu Tang.

**Writing – review & editing:** Feihu Tang, Yong Cui, Yuan Gao.
